# 3,4-Dehydro-L-proline Induces Programmed Cell Death in the Roots of *Brachypodium distachyon*

**DOI:** 10.3390/ijms22147548

**Published:** 2021-07-14

**Authors:** Artur Pinski, Alexander Betekhtin, Jolanta Kwasniewska, Lukasz Chajec, Elzbieta Wolny, Robert Hasterok

**Affiliations:** 1 Plant Cytogenetics and Molecular Biology Group, Institute of Biology, Biotechnology and Environmental Protection, Faculty of Natural Sciences, University of Silesia in Katowice, 40-032 Katowice, Poland; artur.pinski@us.edu.pl (A.P.); jolanta.kwasniewska@us.edu.pl (J.K.); elzbieta.wolny@us.edu.pl (E.W.); 2 Animal Histology and Embryology Group, Institute of Biology, Biotechnology and Environmental Protection, Faculty of Natural Sciences, University of Silesia in Katowice, 40-032 Katowice, Poland; lukasz.chajec@us.edu.pl

**Keywords:** 3,4-dehydro-L-proline (3,4-DHP), *Brachypodium distachyon*, cell wall, cell death, extensin (EXT), hydroxyproline-rich glycoproteins (HRGP)

## Abstract

As cell wall proteins, the hydroxyproline-rich glycoproteins (HRGPs) take part in plant growth and various developmental processes. To fulfil their functions, HRGPs, extensins (EXTs) in particular, undergo the hydroxylation of proline by the prolyl-4-hydroxylases. The activity of these enzymes can be inhibited with 3,4-dehydro-L-proline (3,4-DHP), which enables its application to reveal the functions of the HRGPs. Thus, to study the involvement of HRGPs in the development of root hairs and roots, we treated seedlings of *Brachypodium distachyon* with 250 µM, 500 µM, and 750 µM of 3,4-DHP. The histological observations showed that the root epidermis cells and the cortex cells beneath them ruptured. The immunostaining experiments using the JIM20 antibody, which recognizes the EXT epitopes, demonstrated the higher abundance of this epitope in the control compared to the treated samples. The transmission electron microscopy analyses revealed morphological and ultrastructural features that are typical for the vacuolar-type of cell death. Using the TUNEL test (terminal deoxynucleotidyl transferase dUTP nick end labelling), we showed an increase in the number of nuclei with damaged DNA in the roots that had been treated with 3,4-DHP compared to the control. Finally, an analysis of two metacaspases’ gene activity revealed an increase in their expression in the treated roots. Altogether, our results show that inhibiting the prolyl-4-hydroxylases with 3,4-DHP results in a vacuolar-type of cell death in roots, thereby highlighting the important role of HRGPs in root hair development and root growth.

## 1. Introduction

The plant cell wall is a complex structure, comprising polymers such as cellulose, pectins and hemicelluloses, various aromatic and lipid compounds, and the cell wall proteins. Even though the cell wall proteins constitute less than 10% of the cell wall mass, they play crucial roles in modifying the polymers and in signaling. Among the cell wall proteins, the hydroxyproline-rich glycoproteins (HRGPs) are of primary importance [1,2]. The HRGPs are involved in various plant growth and development processes, such as cell division and differentiation, cell adhesion, somatic embryo development, reprogramming of the cell fate [3], pollen recognition and fertilization [4], programmed cell death (PCD), as well as interacting with beneficial bacteria, such as endophytic bacteria [5,6]. Additionally, changes in the presence of the HRGPs can be linked with the gradual loss of embryogenic potential in callus cultures of *Brachypodium distachyon* [7]. Numerous studies have shown the involvement of the HRGPs in the response to abiotic stresses, such as cold, heat, and salt stress [2,8,9,10]. The HRGPs are usually divided into three complex multigene families: (i) extensins (EXTs), (ii) arabinogalactan proteins (AGPs), and (iii) proline-rich proteins [11]. The EXTs are highly repetitive glycoproteins in which proline residues are hydroxylated by prolyl-4-hydroxylases, which yields hydroxyproline, after which the O-linked glycans are usually attached to the hydroxyl group of hydroxyproline [12,13,14].

PCD is an essential part of plant development and responds to a variety of both abiotic and biotic stresses [15]. DNA breaks are specific features of PCD that can be identified, e.g., using TdT-mediated dUTP nick end labelling, which is more commonly known as the TUNEL test [16,17,18]. Changes in the expression of various genes, such as metacaspases, papain-like Cys proteases, and vacuolar processing enzymes, are other specific hallmarks of PCD that can be analyzed using the RT-qPCR (quantitative reverse transcription PCR) technique [17]. Moreover, the HRGPs are involved in PCD, as was shown by Gao and Showalter [19] using a Yariv reagent, which had been added to *Arabidopsis thaliana* cell suspension cultures. Their involvement was attributed to changes in the AGP localization at the plasma membrane–cell wall interface.

Although identifying the HRGPs is challenging, Johnson, et al. [20] were able to identify the HRGPs in sequences from the 1000 Plant transcriptomes initiative. The main insight of this work was the detection of the loss of the cross-linking EXTs in a few of the lineages, including the grass family (Poaceae). Notably, the cell wall composition in grasses is distinct from those other monocots and eudicots [21]. Therefore, studies that are dedicated to the grass HRGPs are required. Over the years, *B. distachyon* has become a model for cereal crops and temperate grasses because of its small stature, short life cycle, low growth requirements, and relatively small genome. It is worth noting that its reference genome was sequenced and characterized long ago [22,23]. More recent studies have provided whole-genome sequence information regarding the numerous genotypes of *B. distachyon* and its pan-genome, thereby expanding our understanding of the intraspecific variation within this model species [24,25]. Moreover, the protocols for *B. distachyon* and *B. hybridum* transformation and genome editing that have been developed using the CRISPR/Cas9 system are also available [26]. Most importantly, the genes encoding the EXTs and fasciclin-like arabinogalactan proteins that belong to the AGPs are well characterized in *B. distachyon* [27,28].

The importance of the HRGPs for plant growth and development has been studied using various approaches, such as transcriptomic and proteomic analyses, which are often coupled with immunocytochemistry [2,29]. In a few studies, the selective inhibitor of the HRGPs biosynthesis, 3,4-dehydro-L-proline (3,4-DHP), has been used to reveal the function of HRGPs, EXTs in particular [30,31,32,33,34]. 3,4-DHP is a potent inhibitor of prolyl-4-hydroxylase activity at micromole concentrations that results in the rapid and irreversible inactivation of prolyl-4-hydroxylase. Additionally, 3,4-DHP was shown not to be generally toxic, having little effect on the induction of nitrate reductase by nitrate, wound-induced amino acid uptake, and protein synthesis. It was postulated that 3,4-DHP acts as a prolyl hydroxylase enzyme-activated suicide inhibitor [31]. Thus, to study the effects of HRGPs in the germination and root development of *B. distachyon*, we used a wide range of 3,4-DHP concentrations (250 µM, 500 µM, and 750 µM). The morphological observations were combined with histological, immunocytochemical analysis of the selected cell wall epitopes and ultrastructure analyses of the roots. The breaks in the DNA were identified using the TUNEL test. The expression profiles of selected genes involved in PCD were determined using RT-qPCR.

## 2. Results

### 2.1. Impact of 3,4-DHP on B. distachyon Root Development

**Figure 1:**
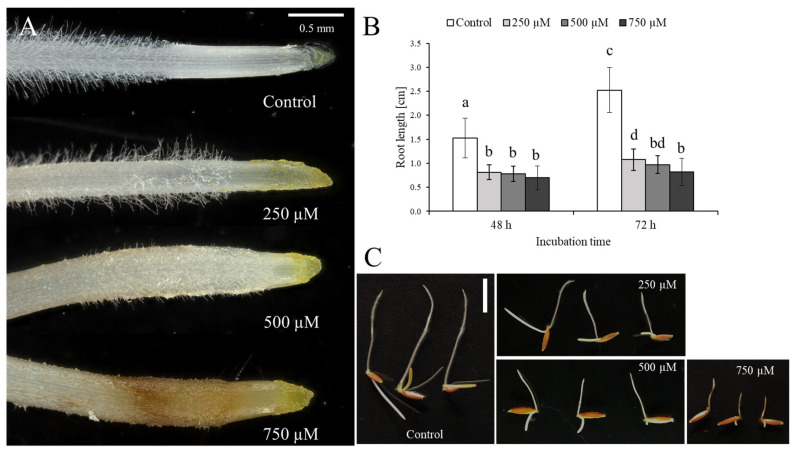
(**A**) Microscopic analysis of the *B. distachyon* root response after treatment with different (250 µM, 500 µM, and 750 µM) concentrations of 3,4-DHP. (**B**) Changes in the root length after 48 h and 72 h of treatment with 250 µM, 500 µM, and 750 μM 3,4-DHP. Error bars indicate the standard deviation (one-way ANOVA followed by Tukey HSD test, *p* < 0.05; mean ± SD, statistically significant differences are indicated by different letters). (**C**) Total root length in the control and after 72 h treatment with 250 μM, 500 μM, and 750 μM 3,4-DHP. Scale bar: 1 cm.

**Figure 2:**
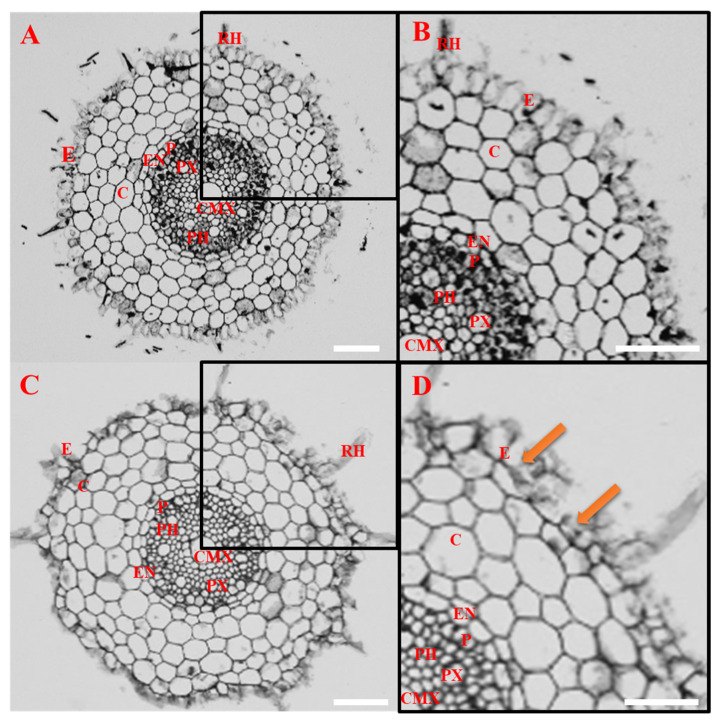
Histological observations of *B. distachyon* root cross-sections in the control (**A**,**B**) and after 72 h of treatment with 750 µM 3,4-DHP (**C**,**D**). Abbreviations: C—cortex, CMX—central metaxylem, E—epidermis, EN—endodermis, P—pericycle, PH—phloem, RH—root hair, and PX—protoxylem. Orange arrows indicate ruptured cells. Scale bars: 50 µm.

**Figure 3:**
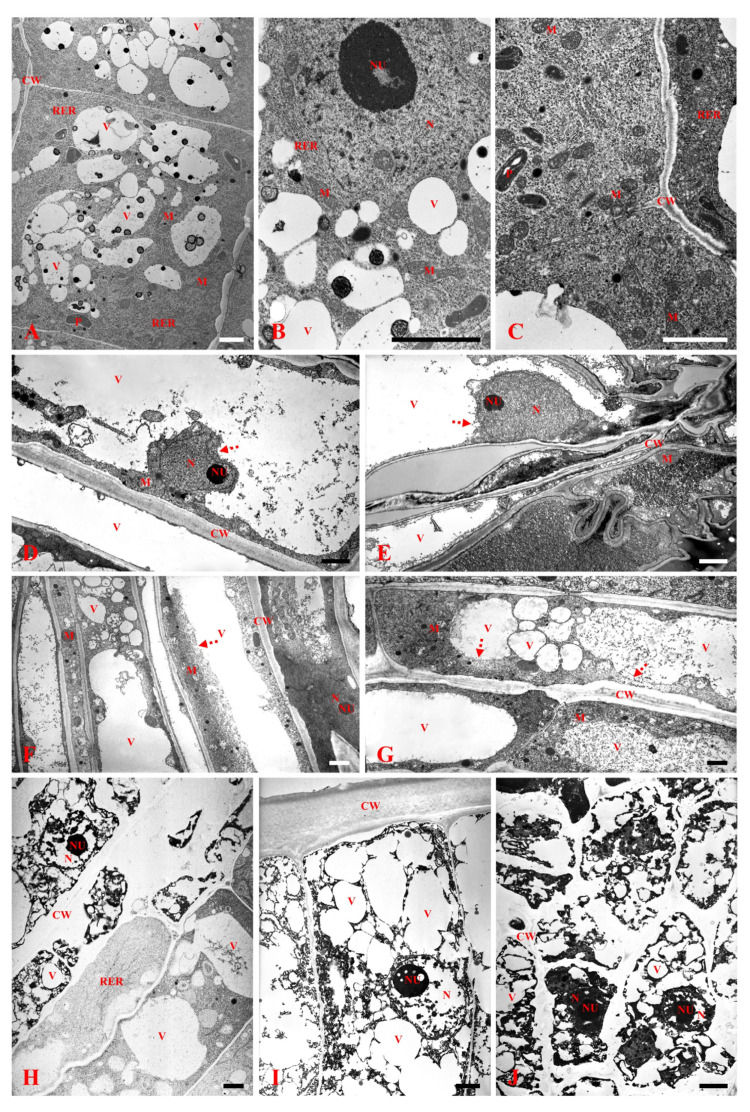
The ultrastructure of roots cells in the control (**A**–**C**) and treated with 250 μM (**D**,**E**), 500 μM (**F**,**G**). and 750 μM (**H**–**J**) of 3,4-DHP. Abbreviations: CW: cell wall; M: mitochondria; N: nucleus; NU: nucleolus; P: plastid; RER: rough endoplasmic reticulum, and V: vacuole. Red arrows demonstrate ruptured tonoplasts. TEM, scale bars: 2 μm.

In order to determine 3,4-DHP’s influence on *B. distachyon* germination and root development, we exposed the seedlings to different (250 µM, 500 µM, and 750 µM) concentrations of 3,4-DHP. After the treatment with 250 µM 3,4-DHP for 72 h, we observed shortened roots tips and a reduced number of root hairs (Figure 1A). After the treatment with 500 µM of 3,4-DHP, its impact was more pronounced and was manifested by shortened root tips and considerably shortened root hairs. After the exposure to the highest concentration (750 µM) of 3,4-DHP, changes in the root morphology were generally similar to those that had been observed for the intermediate concentration; however, extensive root darkening was clearly visible. Tracking the root length, we found that after 48 h, the treatment with 750 µM of 3,4-DHP resulted in around a two-fold shorter root length than in the control (0.7 cm compared to 1.53 cm, respectively (Figure 1B,C)). Interestingly, after 72 h, the root length in the treated seedlings was not significantly different than it was after 48 h of treatment. A similar growth inhibition was observed at the lower concentrations. Notably, the germination efficiency was unaffected by the inhibitor at the applied concentrations. Considering these observations, we decided to closely examine the changes in the internal structure of the roots that had been subjected to the highest (750 µM) concentration of 3,4-DHP. Histological analysis revealed that compared to the control (Figure 2A,B), in the experimental group, rhizodermal cells in the maturation zone were ruptured (Figure 2C,D). In addition, some of the cortex cells in the layer immediately beneath the rhizodermis seemed to be affected by 3,4-DHP (Figure 2D). We further examined the ultrastructure of the rhizodermal cells from roots that had been subjected to the 250 µM, 500 µM, and 750 µM of 3,4-DHP using transmission electron microscopy (TEM, Figure 3). In the control, we were able to clearly identify the cell wall (CW), mitochondria (M), nucleus (N), nucleolus (NU), plastids (P), rough endoplasmic reticulum (RER), and the vacuoles (V) (Figure 3A–C). In the roots that had been treated with 3,4-DHP, we observed significant changes in the surface cells (Figure 3D–J). Treated cells were filled with vacuoles, which were also prominent in the nucleus. Other compartments, such as the mitochondria, plastids, and rough endoplasmic reticulum, were seriously degraded. Although the cell wall was retained in the cells treated with 250 and 500 µM of 3,4-DHP (Figure 3D–G), it was almost completely degraded in the cells treated with 750 µM (Figure 3H–J). Regardless of these massive ultrastructural changes, the nucleolus was still identifiable in the cells. A disruption of the tonoplast preceded the plasma membrane breakdown, making the collapse of the tonoplast the primary executor of the cell death (Figure 3D–J).

### 2.2. The TUNEL Test Demonstrated That DNA Damage Was Induced by 3,4-DHP 

**Figure 4:**
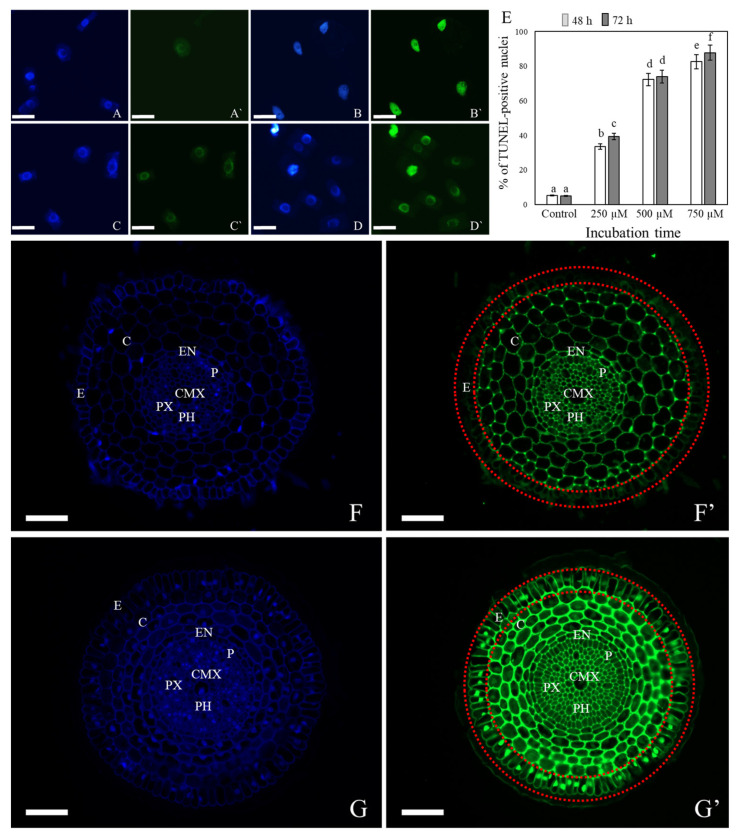
In situ detection of DNA fragmentation in the squashed slides (**A**–**E**) and cross-sections (**F**,**G**) of *B. distachyon* root tips that had been treated with 750 µM of 3,4-DHP using the TUNEL assay. Blue fluorescence: DAPI staining (**A**–**D**,**F**,**G**), green fluorescence: FITC showing the TUNEL-positive nuclei (**A’**–**D’**,**F’**,**G’**); control (**A**,**A’**,**F**,**F’**); positive control (**B**,**B’**); 3,4-DHP treatment for 48 h (**C**,**C’**,**G**,**G’**); 3,4-DHP treatment for 72 h (**D**,**D’**); number of labelled nuclei in the root cells in the control and after 3,4-DHP treatment (**E**). Scale bars: 20 μm (**A**–**D**), 50 μm (**F**,**G**). Statistically significant differences are indicated by different letters (ANOVA followed by the Tukey HSD test, *p* < 0.05; mean ± SD). Abbreviations: C—cortex, CMX—central metaxylem, E—epidermis, EN—endodermis, P—pericycle, PH—phloem, and PX—protoxylem. Epidermis is marked with two red dotted circles.

The TUNEL test was used to analyze the number of nuclei with DNA breaks in the root maturation zone of the control plants and the plants that had been treated with 3,4-DHP for 48 h and 72 h. All of the cells were counterstained with DAPI to determine the percentage of damaged nuclei. The nuclei that had a green fluorescence in the FITC channel were characterized by DNA damage (Figure 4A–E). The material that had been treated with DNase showed TUNEL-positive signals in 94% of the nuclei for the positive control. No FITC-labelled nuclei were observed in the negative control. The analysis revealed that the frequency of TUNEL-positive nuclei did not change significantly in the control roots at the 48 h (5.2%) and 72 h (6.5%) time points. The frequency of TUNEL-positive nuclei increased significantly in the roots that had been treated with 3,4-DHP in a dose-dependent manner and were 33.5% at a concentration of 250 µM, 72.3% at 500 µM, and 82.6% at 750 µM after 48 h (Figure 4E). The frequency of the TUNEL-positive nuclei slightly increased after 72 h, compared to the previous time-point. To show in detail which tissues were the most affected by the inhibitor, the TUNEL test was performed on root cross-sections. The nuclei with damaged DNA were present mainly in the rhizodermis (Figure 4F,G). 

### 2.3. Localisation of EXT Epitopes under Different 3,4-DHP Treatments

**Figure A1:**
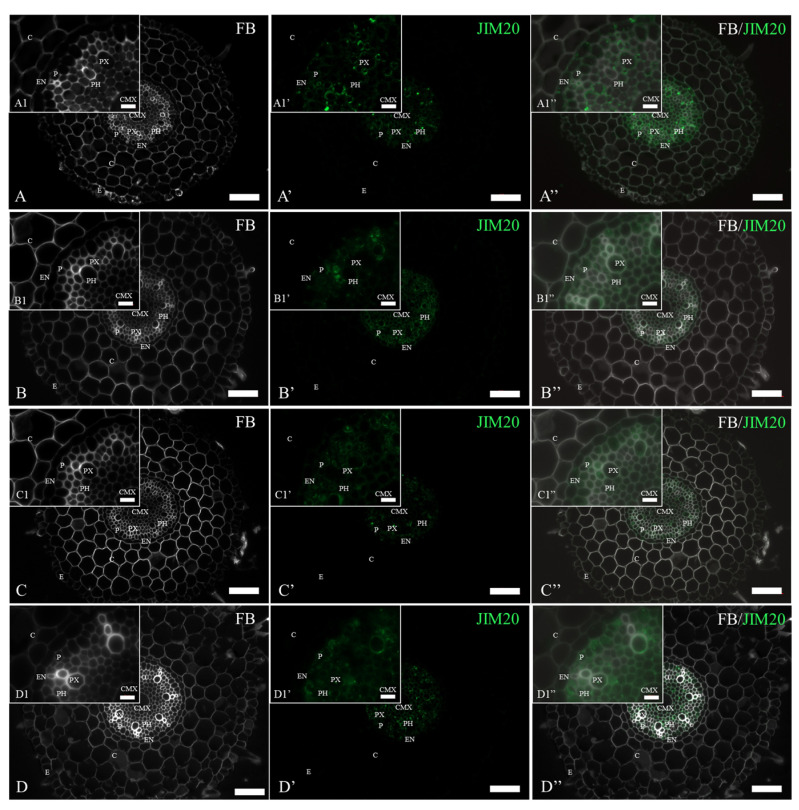
Immunolocalization of the JIM20 epitope (**A**–**D**) in cross-sections of the *B. distachyon* roots, (**A**,**A’**,**A”**): the control; (**B**,**B’**,**B”**): 250 µM 3,4-DHP; (**C**,**C’**,**C”**): 500 µM 3,4-DHP, (**D**,**D’**,**D”**): 750 µM 3,4-DHP. Abbreviations: C—cortex, CMX—central metaxylem, E—epidermis, EN—endodermis, FB—fluorescent brightener, P—pericycle, PH—phloem, and PX—protoxylem. The green color shows epitope occurrence. Scale bars: 10 μm for A1–C1, while for the remaining photomicrographs, 50 μm.

**Figure A2:**
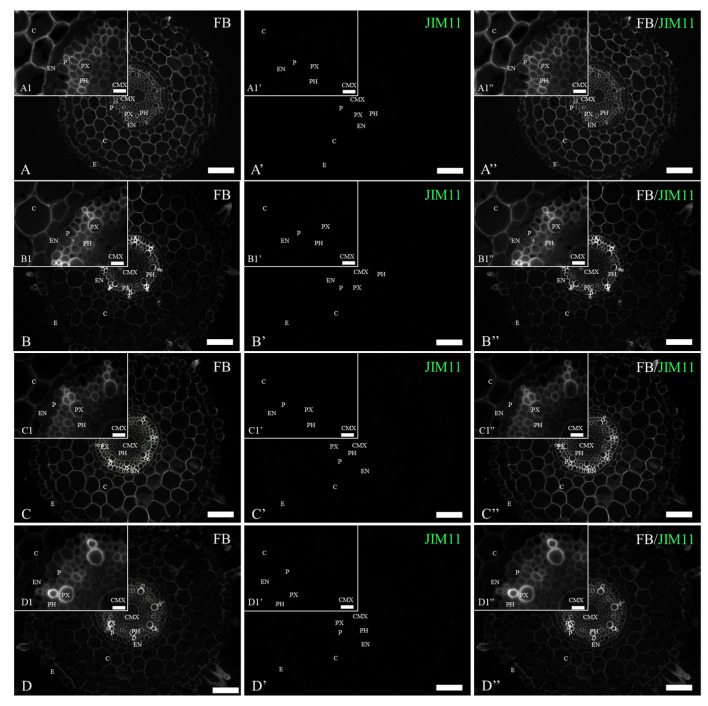
Immunolocalization of the JIM11 epitope (**A**–**D**) in cross-sections of the *B. distachyon* roots, (**A**,**A’**,**A”**): the control; (**B**,**B’**,**B”**): 250 µM 3,4-DHP; (**C**,**C’**,**C”**): 500 µM 3,4-DHP, (**D**,**D’**,**D”**): 750 µM 3,4-DHP. Abbreviations: C—cortex, CMX—central metaxylem, E—epidermis, EN—endodermis, FB—fluorescent brightener, P—pericycle, PH—phloem, and PX—protoxylem. The green color shows epitope occurrence. Scale bars: 10 μm for A1–C1, while for the remaining photomicrographs, 50 μm.

To test whether the selected EXT epitopes were different between the control and material treated with 3,4-DHP, immunocytochemical analyses were performed using specific monoclonal antibodies (JIM11 and JIM20). The EXT epitopes recognized by the JIM20 antibody were observed mainly in the root vascular bundle (central metaxylem, endodermis, pericycle, phloem, and protoxylem). In the control samples, the signal was detected primarily in the cell wall, while in the treated root, signal was present in the cell walls and in the intercellular compartments (which we define as the localization of the epitope within the cytoplasm endomembrane system or organelles that are associated with the biosynthesis and secretion pathway to the wall [35]). The fluorescence signal from this epitope was more abundant in the control than in the treated samples (Figure A1). No signal for the JIM11 epitope was detected in the roots cross-sections (Figure A2). 

### 2.4. Changes in the Expression of EXTs and Genes Associated with PCD 

**Figure 5:**
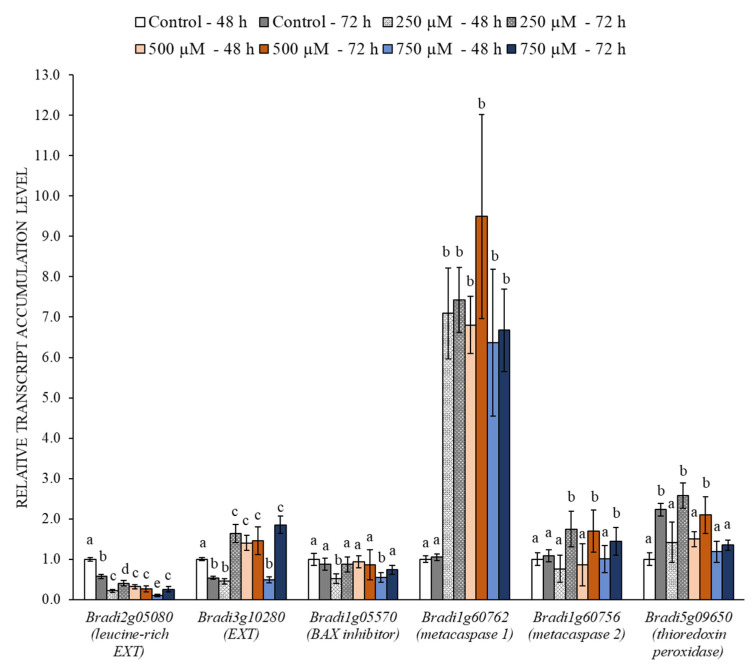
The relative level of transcript accumulation of EXTs and PCD-related genes. The relative expression levels were normalized to an internal control (*AK437296*, gene encoding ubiquitin). Statistically significant differences are indicated by different letters (ANOVA followed by the Tukey HSD test, *p* < 0.05; mean ± SD).

We measured the changes in the expression levels of the genes associated with PCD in the control and in the roots that had been treated with 3,4-DHP. These included two genes encoding the metacaspases (*Bradi1g60762* and *Bradi1g60756)* and one gene encoding the *BAX inhibitor* (*Bradi1g05570*). The genes were selected as was described previously [17]. We considered both time points, i.e., after 48 h and 72 h (Figure 5). Focusing on the responses to the inhibitor, we observed an increase in the expression of all of the genes, although the increase in the expression of genes encoding metacaspases was more prominent and progressed over the incubation time. *Bradi1g60762* (*metacaspase 1*) had an around eight-fold increase in its expression in response to 3,4-DHP in all tested concentrations. In contrast, the expression of this gene remained unchanged in the control at both analyzed time-points. For *Bradi1g60756* (*metacaspase 2*), we observed around a twofold increase in its expression in response to the 3,4-DHP treatment after 72 h. However, the expression 48 h in these treatments remained the same as in the control. For both incubation times and all treatments, the *BAX inhibitor* gene showed a similar expression pattern, being mostly unchanged. Additionally, we observed a mostly unaffected expression of the *thioredoxin peroxidase* gene, being similar to the control at the respective time-points (Figure 5). The expression of the two genes encoding EXTs differed in the analyzed treatments. The *Bradi2g05080* gene showed a decreased expression in response to 3,4-DHP, with expression being up to 7.7-fold lower in the highest analyzed concentration after 72 h (Figure 5). The second gene encoding EXT (*Bradi3g10280*) showed around a twofold increase in the expression in response to the 3,4-DHP treatment. 

## 3. Discussion

The HRGPs were shown to be essential for plant growth and developmental processes, as was revealed in various experiments using 3,4-DHP, which is an inhibitor of the prolyl-4-hydroxylases. For example, Bucher, et al. [32] observed a reduction in the root length in tomato by almost 50% at a 10 µM concentration of 3,4-DHP. These authors reported a further shortening of the roots at higher tested concentrations, with the highest reduction occurring at the 100 µM concentration, where the roots were 5.6-fold shorter than those in the control. For *B. distachyon*, we observed twofold shorter roots at all tested concentrations compared to the control, which most likely indicates a species-specific response to 3,4-DHP. Interestingly, similar to our experiment, seed germination in tomato was not influenced by 3,4-DHP and was the same as in the control treatment [32]. In banana somatic embryos, 3,4-DHP at a concentration of 200 µM inhibited the development of embryogenic cells and decreased the embryo germination rate, which finally led to a decreased regeneration capacity. Notably, the treatment also resulted in an aberrant non-compact epidermis with a discontinuous extracellular matrix [33]. In our experiment, the root epidermis and cortex cells that were directly beneath the root epidermis were the most affected ones, supposedly because, as the most externally localized, they are particularly susceptible to the action of an inhibitor. In another experiment, 3,4-DHP at a concentration of 200 µM was shown to inhibit the symbiotic germination of orchid seeds [34]. Treatment with 3,4-DHP at a range of concentrations (from 10 to 200 µM) slowed the growth speed of the pollen tube of *Nicotiana tabacum*. It was also indicated that the inhibitor affected the elongation of style cells without any relevant influence on cell division [36]. Using immunocytochemistry experiments with a JIM20 antibody, we have shown that a 3,4-DHP inhibitor has a direct effect on EXTs presence. Treatment with an inhibitor resulted in the decreased abundancy of the epitope and changes in the signal distribution. A fluorescence signal from this epitope, mainly in the intercellular compartments, was observed. It should be noted that banana somatic embryos treated with 3,4-DHP resulted in the depletion of the surface-localized epitopes recognized by JIM11 and JIM20 and, subsequently, decreased plant regeneration capacity in embryogenic banana cultures. [33,37]. Similarly, a lower abundancy of the epitope recognized by JIM20 after treatment with 3,4-DHP was observed in styles of *N. tabacum* [36]. As we have shown, treatment with 3,4-DHP also decreases the expression of genes encoding leucine-rich EXT (*Bradi2g05080*). Opposite to this leucine-rich EXT, another EXT gene (*Bradi3g10280*) increased its expression in the material treated with 3,4-DHP after 72 h. Such changes in the EXT gene expressions seem to be connected with the activation of the cell death processes [38]. 

In *A. thaliana*, the prolyl-4-hydroxylases (*AtP4H1*) have been shown to hydroxylate the proline-rich peptides in vitro. The overexpression lines of *AtP4H1* plants had a hypoxia-in-normoxia phenotype that was accompanied by an increased number of root hairs, the absence of trichomes, and a reduced seed size, which indicates the involvement of *AtP4H1* in hypoxia stress, as well as in the different stages of plant growth and development [39]. We demonstrated that when the prolyl 4-hydroxylase inhibitor was applied at a 250 µM concentration, it caused changes in the root hair development. In comparison, higher concentrations (500 µM or 750 µM) caused a reduction in root hair length. In tomato, the suppression of prolyl-4-hydroxylases resulted in a delay of the abscission progression in overripe tomato fruits 90 days after the breaker stage. These changes were linked with the downregulation of the expression of the cell wall hydrolases, cellulases, and expansins [40]. Other studies have shown that three prolyl 4-hydroxylase inactivated mutants had an apparent short root hair phenotype and reduced root hydroxyproline levels. Conversely, the overexpression of these P4Hs resulted in increased root hair length and density. Treatment with α,α-dipyridyl and ethyl-3,4-dihydroxybenzoate, which inhibit the activity of prolyl-4-hydroxylases, resulted in the inhibition of root hair elongation [41,42]. Moreover, in our experiment, treatment with 3,4-DHP resulted in shortened root hairs, especially after 750 µM of 3,4-DHP was applied. There is an abundance of research that underlines the involvement of HRGPs in root hair development. For example, a mutant of *A. thaliana* in gene-encoding chimeric leucine-rich repeat/extensin protein (LRX1) that was inactive developed root hairs that frequently branched, swelled, or were aborted [43]. Likewise, a global analysis of the root hair transcriptome revealed new candidate genes that are involved in root hair formation in barley, including these coding for AGPs, EXTs, and leucine-rich-repeat proteins [44]. These and other research suggest that the proline hydroxylation of HRGPs, EXTs in particular, is essential for polarized cell expansion in root hairs [42,45]. Proline hydroxylation is followed by O-glycosylation, which stabilizes the EXT short peptide’s helical conformation, while incomplete O-glycosylation increases its flexibility. Notably, the proline hydroxylation of EXTs is absolutely required for the subsequent O-glycosylation stages. Thus, it was postulated that an incomplete hydroxylation/O-glycosylation of EXTs impacts their interactions with other EXTs and with the peroxidases that are involved in the EXTs cross-linking [41]. Indeed, two class III peroxidase-encoding genes were identified as being essential for the correct anther and pollen development in *A. thaliana*. These peroxidases contribute to the integrity of the tapetal cell wall during anther development, most likely due to the cross-linking of EXTs [46]. Furthermore, the degree of hydroxyproline-O-arabinosylation of EXTs also affects the degree of tyrosine cross-linking [38,47]. Additionally, the correct O-glycosylation is required for the appropriate targeting of proteins, as was shown in AGP21 [48]. These postulates regarding EXTs cross-linking contradict our observations of the impact of 3,4-DHP on root hair development in *B. distachyon*, which, like other grasses, is devoid of the cross-linking EXTs [20].

The histological observations combined with an ultrastructure analysis of the roots that had been treated with 3,4-DHP identified PCD of the root epidermis cell. Furthermore, the ultrastructure analysis showed the disruption of the compartments with a vacuolization that altogether suggest a vacuolar type of cell death [15]. It was demonstrated that this type of cell death occurs naturally during embryo development, tissue and organ differentiation, and in response to stress conditions [15,49,50]. The TUNEL test is widely used to investigate the presence of DNA double-strand breaks, a specific feature of PCD [51,52]. For example, this test was used to visualize DNA fragmentation in *B. distachyon* embryogenic callus cells that had been treated with 5-azacitidine [17]. In our work, we demonstrated damage to the DNA as a result of the treatment with 3,4-DHP using the TUNEL test. The frequency of TUNEL-positive nuclei was found to be dose-dependent, showing 39% after 72 h at concentration 250 µM and reaching 86% after 72 h at concentration 750 µM. Moreover, the number of nuclei with damaged DNA increases with treatment time. It is worth noting that in the control, we observed TUNEL-positive nuclei at a frequency of 6.5%, which aligns with the well-known fact that to some extent DNA double-strand breaks can be spontaneously generated [53]. As was revealed by the TUNEL test, the genomic DNA breaks suggest that PCD occurs in the roots, which we further confirmed by analyzing the expression levels of the genes encoding metacaspases [17,54]. In plants, metacaspases play essential roles in PCD, specifically in processes such as signalling, developmental regulation, and stress-induced PCD [55,56,57]. We observed an increased expression of both of the studied metacaspases in response to the treatment with 3,4-DHP at all tested concentrations. A similar increase in metacaspase expression was observed in tomato leaves when infected by *Botrytis cinerea*, which is a fungal pathogen that induces cell death in several plant species [58]. We can distinguish two classes of plant cell death that have distinct kinetics and morphology: necrosis and vacuolar cell death. While necrosis is a rapid process that involves mitochondrial dysfunction and early loss of plasma membrane integrity, vacuolar cell death is a slow process in which the growing lytic vacuoles gradually digest cells. Metacaspases regulate the switch between vacuolar cell death and necrosis, as is shown by their genetic suppression, which causes a toggling from one kind of the cell death to another [59]. Thus, metacaspases can be seen as being executors of vacuolar cell death [60]. Similar to the control, *Bradi5g09650* (encodes thioredoxin peroxidase) expression levels suggest no oxidative stress induced by the inhibitor in the root cells [61]. 

As demonstrated earlier, the absence of HRGPs induced by 3,4-DHP treatment resulted in the developmental arrest and death of protocorms, attributed to the alteration of the internal regulatory processes [34]. It was previously postulated that AGPs and EXTs might be involved in cell death [19,38,62,63]. In *A. thaliana*, it was shown that different prolyl-4-hydroxylases exhibit diverse tissue expression profiles, subcellular localization, and substrate preference. Thus, even a triple *A. thaliana* mutant *p4h2*,*5*,*13* was not more affected in the root hairs growth than a single *p4h5* mutant showing redundancy of prolyl-4-hydroxylases. Furthermore, until now, no mutants in prolyl-4-hydroxylases were shown to be lethal [64,65]. Since 3,4-DHP is a selective inhibitor of prolyl-4-hydroxylases, inactivation of all of them may result in significant changes in the cell wall structure, which may lead to cell death. Furthermore, it was shown that AGPs are markers of cell death during microsporogenesis in *A. thalina* [66]. It is possible that alterations in the cell wall structure may lead to the induction of the cell death, especially as it was shown that the cell wall structure participates in the transduction of signals between the cells [67,68]. 

## 4. Materials and Methods

### 4.1. Plant Material and 3,4-DHP Treatment

The seeds of the *B. distachyon* genotype Bd21 (accession number: PI 254867) were sourced from the collection held by the United States Department of Agriculture, National Plant Germplasm System. They were germinated on three layers of filter paper that had been soaked with distilled water or the treatment solution in Petri dishes. The plants were grown in the dark at 21 ± 1 °C. The 200 mM aqueous stock solution of 3,4-DHP was prepared and added to the distilled water in order to obtain a final concentration of 250 µM, 500 µM, or 750 µM. The material for the analyses were collected after 48 h and 72 h of the control and experimental treatment. For calculating the root length and germination efficiency, at least 40 seedlings in three biological replications were included. The root length was measured using ImageJ v. 1.52 (NIH and Loci, University of Wisconsin, Madison, WI, USA).

### 4.2. Histological Procedures 

The procedures for embedding the tissues in Steedman’s wax [69] and preparing the slides were done according to Wolny, et al. [70]. The roots were fixed in a mixture of 4% (*w*/*v*) paraformaldehyde and 1% (*v*/*v*) glutaraldehyde in phosphate-buffered saline (PBS, pH 7.0) overnight at 4 °C. Then, the roots were rinsed with PBS (3 × 15 min) and dehydrated in an ascending ethanol series (10%, 30%, 50%, 70%, 90%, and 100%; 2 × 30 min in each). For the toluidine blue staining (Sigma-Aldrich, St. Louis, MO, USA), slides with the tissue sections were de-embedded 3 × for 10 min in 99.8% ethanol and rehydrated in ethanol/distilled water for 10 min at each step (90%, 70%, 50%, 30% *v*/*v*, distilled water). The slides were then placed in an aqueous 0.01% toluidine blue solution for 10 min and rinsed three times in distilled water for 5 min each. The stained slides were then air-dried and embedded in a mounting medium (DPX, Sigma-Aldrich, St. Louis, MO, USA). Images of the stained tissue sections were obtained using an Axio Imager Z2 microscope equipped with an AxioCam camera (Zeiss, Oberkochen, Germany).

### 4.3. RT-qPCR

To characterize the level of the transcript accumulation of the genes associated with the cell death and EXT, RT-qPCR was performed according to the detailed procedure described by Betekhtin, et al. [17]. Briefly, the total RNA was isolated from the whole roots of *B. distachyon* using the protocol described by Muoki, et al. [71] using buffers containing cetrimonium bromide and phenol. The isolated RNA was run on a 1% agarose gel to check for quality and integrity. The good-quality RNA was treated with DNase for 10 min at room temperature (Qiagen, Hilden, Germany) and then used for first-strand cDNA generation with oligo(dT) primers (Maxima First Strand cDNA Synthesis Kit, Thermo Fisher Scientific, Waltham, MA, USA). The primers used in this research were previously described in [17] and are presented in Table A1. The samples were run using a LightCycler^®^ 480 Real-Time PCR System (Roche, Basel, Switzerland). The qPCR conditions were as follows: 5 min at 95 °C, 45 cycles of 10 s at 95 °C, 20 s at 60 °C, and 10 s at 72 °C with the signal acquisition. The *AK437296* gene coding for ubiquitin was used as the reference, and the analysis was performed using the 2^−∆∆CT^ method [72]. The significant differences among samples were analyzed using ANOVA and evaluated using a post hoc Tukey HSD test. 

### 4.4. TUNEL Assay

The TUNEL assay was performed as was previously described [51]. The root tissue was fixed with 4% (*w*/*v*) paraformaldehyde for 1 h at room temperature and then washed 3× for 5 min in PBS. The squashed slides with the nuclei and roots cross-section slides were prepared in PBS, frozen at −70 °C, and finally, air-dried at room temperature. The slides were then incubated in a permeabilization solution (0.1% Triton X-100 in 0.1% sodium citrate) for 2 min at 4 °C and rinsed with PBS. The positive control was made for the squashed slides by adding 50 µL of a DNase solution (250 µg/mL) to a slide with the control material for 30 min at 37 °C in a humid chamber. After the DNase treatment, the slides were rinsed twice with PBS. The DNA fragments were labelled using the TUNEL reaction mixture (In Situ Cell Death Detection Kit, Fluorescein, Sigma-Aldrich, St. Louis, MO, USA). A total of 50 µL of the TUNEL reaction mixture (enzyme: fluorescein-labelled nucleotides, 1:9 ratio, *v*/*v*) was applied on the slides, which were then incubated in a humid chamber in the dark for 1 h at 7 °C. To prepare the negative control, 50 µL of the reaction mixture without terminal transferase was used. The slides were rinsed 3× with PBS, counterstained with DAPI (2 µg/mL), and mounted in Vectashield (Vector Laboratories, Peterborough, UK). The slides were observed using an Axio Imager Z2 wide-field fluorescence microscope equipped with an AxioCam Mrm monochromatic camera and the appropriate sets of filters (Zeiss, Oberkochen, Germany). The number of labelled nuclei was estimated based on an analysis of at least 500 cells from two squashed slides for each treatment.

### 4.5. TEM 

All of the root samples were fixed in 2.5% glutaraldehyde in a 0.1 M sodium phosphate buffer (pH 7.4) for 24 h at 4 °C. After washing in the phosphate buffer, the material was post-fixed in 1% osmium tetroxide in a 0.1 M phosphate buffer (2 h, 4 °C), rinsed with the same buffer, dehydrated in a graded series of ethanol and acetone, and infiltrated successively through mixtures of acetone and Epon 812 resin (3:1, 1:1 and 1:3). Next, the material was embedded in Epon 812 resin (Fullam, Latham, NY, USA). Ultra-thin sections (70 nm thick) were cut on an Ultracut UCT25 ultramicrotome (Leica, Wetzlar, Germany) and collected on copper grids (300 mesh, Electron Microscopy Science, Hatfield, PA, USA). The sections were stained with uranyl acetate and lead citrate and examined using a Hitachi H500 TEM (Hitachi, Tokyo, Japan) at 75 kV.

### 4.6. Immunohistochemistry

The detailed procedure for immunochemical analysis was previously described [73]. The slides were stained with 0.01% (*w*/*v*) fluorescent brightener 28 (FB) (Sigma-Aldrich, St. Louis, MO, USA) in PBS for visualization of the cell walls. Two biological replicates were performed, with at least eight sections for each replicate. To determine the presence of the EXT epitope, JIM20 and JIM11 antibodies (Plant Probes, Leeds, UK) were used [74]. The slides were observed using an Axio Imager Z2 wide-field fluorescence microscope equipped with an AxioCam Mrm monochromatic camera and the appropriate sets of filters (Zeiss, Oberkochen, Germany).

## 5. Conclusions

We demonstrated that 3,4-DHP, an inhibitor of prolyl 4-hydroxylase, causes vacuolar cell death in the roots of *B. distachyon*. Our study highlights the importance of HRGPs in root hair development and root growth, and it shows the need for further research into the function of HRGPs in grasses. 

## Data Availability

The data presented in this study are available on request from the corresponding authors. The data are not publicly available as they are contained in laboratory notebooks.

## Abbreviations

3,4-DHP	3,4-dehydro-L-proline
AGPs	Arabinogalactan proteins
EXTs	Extensins
HRGPs	Hydroxyproline-rich glycoproteins
PBS	Phosphate-buffered saline
PCD	Programmed cell death
RT-qPCR	Quantitative reverse transcription PCR
TEM	Transmission Electron Microscopy
TUNEL	Terminal deoxynucleotidyl transferase dUTP nick end labelling

## Appendix A

**Table A1:** The oligonucleotide primers that were used for the RT-qPCR reaction with relevant descriptions of the genes.

Genes	Description of the Genes	Primer Sequence (5′-3′)
*Bradi1g32860*	*ubiquitin*	pF—GAGGGTGGACTCCTTTTGGA
		pR—TCCACACTCCACTTGGTGCT
*Bradi2g05080*	*leucine-rich EXT*	pF—CTCCGGTTCAACGAGTTCGAG
		pR—CGATGTTATCCGGGAGGTTGAA
*Bradi3g10280*	*EXT*	pF—CTCGTCAGCCGGACATGATA
		pR—TCATGGGGATTTGGACCACG
*Bradi1g05570*	*BAX inhibitor*	pF—ACGCCATCGTCCTGATGTTGTTC
		pR—TGAGGAAGGCCGAGAAGATGAGC
*Bradi1g60762*	*metacaspase 1*	pF—ACTGCATCCTCATCCTCACAGAG
		pR—AGCCAGCAGATTCTCCTTCGTC
*Bradi1g60756*	*metacaspase 2*	pF—ACTGCATCCTCACCCTTACACC
		pR—AGAAGTGGAACACCAGGGAGTC
*Bradi5g09650*	*thioredoxin peroxidase*	pF—GAACCCTTCAGGCCCTGCAATATG
		pR—AACCTGCTGGGCAAACCTCATC
